# The TAZ–miR-224–SMAD4 axis promotes tumorigenesis in osteosarcoma

**DOI:** 10.1038/cddis.2016.468

**Published:** 2017-01-05

**Authors:** Jianjun Ma, Kangmao Huang, Yan Ma, Menglu Zhou, Shunwu Fan

**Affiliations:** 1 Department of Orthopaedic Surgery, Sir Run Run Shaw Hospital, Medical College of Zhejiang University, Sir Run Run Shaw Institute of Clinical Medicine of Zhejiang University, Hangzhou, Zhejiang Province, China; 2 Institute of Biochemistry, College of Life Science, Zhejiang University, Hangzhou, Zhejiang Province, China

## Abstract

Transcriptional co-activator with PDZ-binding motif (TAZ) is a downstream effector of the Hippo signaling pathway that participates in tumorigenesis. The aim of this study was to identify the miRNA counterpart for TAZ and elucidate the mechanism underlying the tumorigenic effect of TAZ. We demonstrated that TAZ is upregulated in osteosarcoma (OS) tissues and cell lines, and that TAZ overexpression can induce cell migration, invasion and proliferation. Moreover, miRNA-224 (miR-224), a TAZ phenocopy that functions downstream of TAZ, was found to be upregulated with TAZ overexpression. Further, a mechanistic study revealed that miR-224 functions by inhibiting the tumor suppressor, SMAD4, to support the proliferation and migration of OS cells. Our findings indicate that targeting TAZ and miR-224 could be a promising approach for the treatment of OS.

Osteosarcoma (OS) is the most common primary malignant bone tumor diagnosed in young people below the age of 20. Formerly, patients with only localized disease were treated with surgery alone, leading to a low survival rate of 20%. This may be attributed to overlooking the micrometastasis likely present at diagnosis.^1^ Nowadays, the 5-year survival rate has risen to approximately 60–70%, owing to cooperative treatment of localized tumor with neoadjuvants.^2^ However, the 5-year survival rate of patients with metastatic tumor remains low at only 30%.^3^ In addition, valid chemotherapy has been constrained by the development of chemoresistance, especially multi-drug resistance, after continuous treatment. Under these circumstances, further refinement with cytotoxic chemotherapy regimens is unlikely.^4^ Hence, novel therapeutic approaches based on targeting oncogenic driver pathways in OS are needed urgently.

The Hippo signaling pathway has a critical role in organ size control, tissue homeostasis and tumorigenesis,^5, 6^ and dysregulation of the Hippo pathway exerts significant impact on malignant transformation.^7^ Mst1/2 are pro-apoptotic kinases and core components of the Hippo pathway; Mst1/2 are activated by caspase-mediated cleavage upon apoptotic stress. Subsequently, Lats1/2 are activated and phosphorylated.^8^ The major downstream effectors of the Hippo pathway are Yes-associated protein (YAP) and transcriptional co-activator with PDZ-binding motif (TAZ),^8^ which can be phosphorylated and inactivated by Lats1/2 in complex with the scaffold protein Mob1.^8, 9, 10, 11^ As transcription co-activators, YAP/TAZ activate gene expression by interacting with the TEAD family of transcription factors.^12, 13, 14^ Increasing evidence has confirmed the aberrant expression of TAZ in multiple human tumors, including breast cancer, lung cancer and hepatocellular carcinoma.^15, 16, 17^ Moreover, it has been demonstrated that TAZ can function as an oncogene, activating TEAD-mediated gene transcription to promote tissue growth and inhibit apoptosis.^15, 16^ However, the downstream function of YAP/TAZ in the Hippo signaling pathway remains unclear.

MicroRNAs (miRNAs) are a class of 22-nucleotide noncoding RNAs that have emerged as critical components of the gene regulatory networks controlling numerous important pathophysiological processes, including the initiation and progression of cancers. Dysregulated miRNA has been demonstrated to have critical roles in OS.^18, 19^ Shen *et al.*^6^ reported that miR-130a is directly induced by YAP, leading to the amplification of YAP activity. A similar mechanism has been reported in the case of Bantam – a well-known Yki-induced miRNA in *Drosophila*.^20, 21^ Moreover, YAP has been identified as a regulator of global miRNA biogenesis via modulation of the miRNA-processing enzymes, microprocessor or Dicer complex, suggesting a transcription-independent role for YAP.^22, 23^

Tumor growth factor (TGF)-*β* has a confirmed yet complicated role in directing the autonomous, local and systemic cellular responses that together regulate the initiation, progression and prognostic outcome of human cancers.^24, 25, 26^ Other pathways altered in human cancer may also contribute to the TGF-*β*-mediated regulation of tumorigenesis to some extent.^27, 28^ During initiation and early progression of the tumor, TGF-*β* serves as a tumor suppressor by inhibiting proliferation and accelerating apoptosis, as evidenced by the fact that loss or mutation of the members of the TGF-*β* signaling pathway in humans causes unregulated cell growth and eventually, cancer. SMADs are key intracellular mediators of transcriptional responses to TGF-*β*. SMAD4 is the pivotal factor of the TGF-*β* pathway and functions as a key tumor suppressor. Dysregulated SMAD4 expression has been reported in some cancers, including OS.^29^

In this study, we aimed to identify the miRNA counterpart of TAZ and elucidate the mechanism underlying the effect of TAZ in OS. We found that TAZ expression is upregulated in OS tissues. Through miRNA sequencing, we investigated the potential roles of TAZ and its related target miRNA-224 (miR-224) in OS development. Then, we demonstrated that TAZ and miR-224 are tumor promoters that accelerate OS progression by promoting cell growth, invasion, tumorigenesis and metastasis. In addition, SMAD4 was identified as a pro-tumorigenic gene and a direct functional target of miR-224 in OS. This study, for the first time, demonstrated the pro-OS effect of TAZ and miR-224, both *in vitro* and *in vivo*. Our results may provide the basis for a novel treatment and diagnosis strategy for TAZ-upregulated OS.

## Results

### TAZ expression was higher in OS patient tissues than in chondroma-innocent tumor tissues

**Figure 1:**
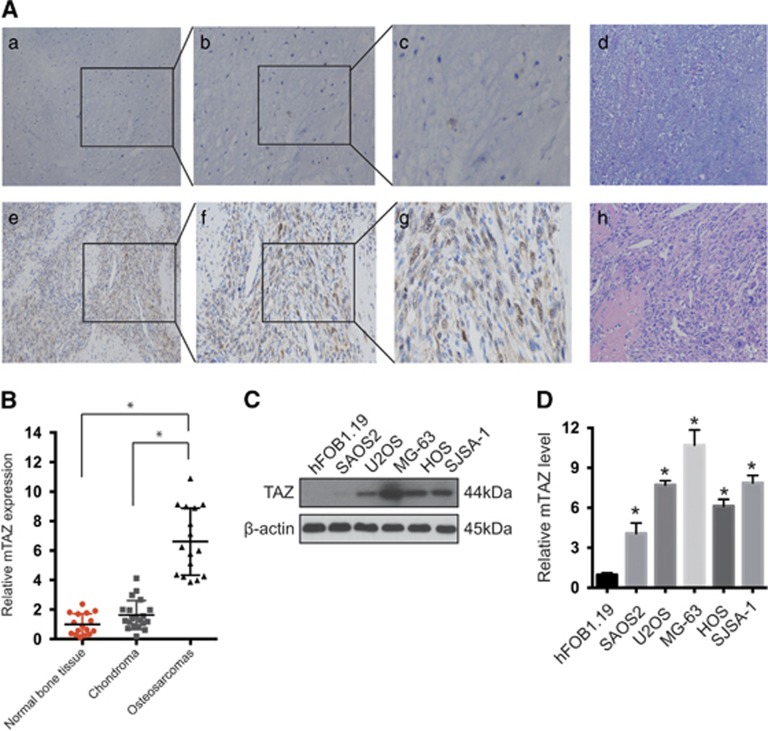
High expression of TAZ in OS. TAZ expression was higher in human OS tissues than in chondroma tissues. (**A**) TAZ staining in OS (e–g) and chondroma tissues (a–c). Magnifications: × 100 (a, e), × 200 (b, f) and × 400 (c, g). Representative results are shown. The areas that were magnified as panels b, c, f and g were marked in panels a, b, e and f, respectively. (d and h) Represent hematoxylin and eosin (H&E) staining of chondroma tissues and OS tissues, respectively. (**B**) TAZ mRNA expression was higher in human malignant OS tissues than human chondroma-innocent tumor tissues. (**C**) TAZ protein expression was higher in human OS cell lines than in non-transformed human osteoblastic cells. (**D**) TAZ mRNA expression was higher in human OS cell lines than in non-transformed human osteoblastic cells (*n*=4, mean±S.D., *P<0.01)

Expression of TAZ was detected in OS and control tissues. In most human chondroma-innocent tumor tissues (90%, 18/20), only a low level of TAZ or vacant labeling was detected (Figure 1A (a–c)). In contrast, strong staining for TAZ was observed in most of the OS tissues (81.25%, 13/16; Figure 1A (e–g)). Relative TAZ mRNA expression was significantly higher in OS tissues than in chondroma tissues or normal bone tissue (*P*<0.01; Figure 1B). Consistent with these observations, OS cell lines U2OS, SAOS2, HOS, SjSA-1 and MG-63 were found to express higher levels of TAZ protein and mRNA relative to normal human osteoblastic cell line, as measured by western blotting and real-time PCR (Figures 1C and D).

### Inhibition of TAZ represses tumorigenesis

**Figure 2:**
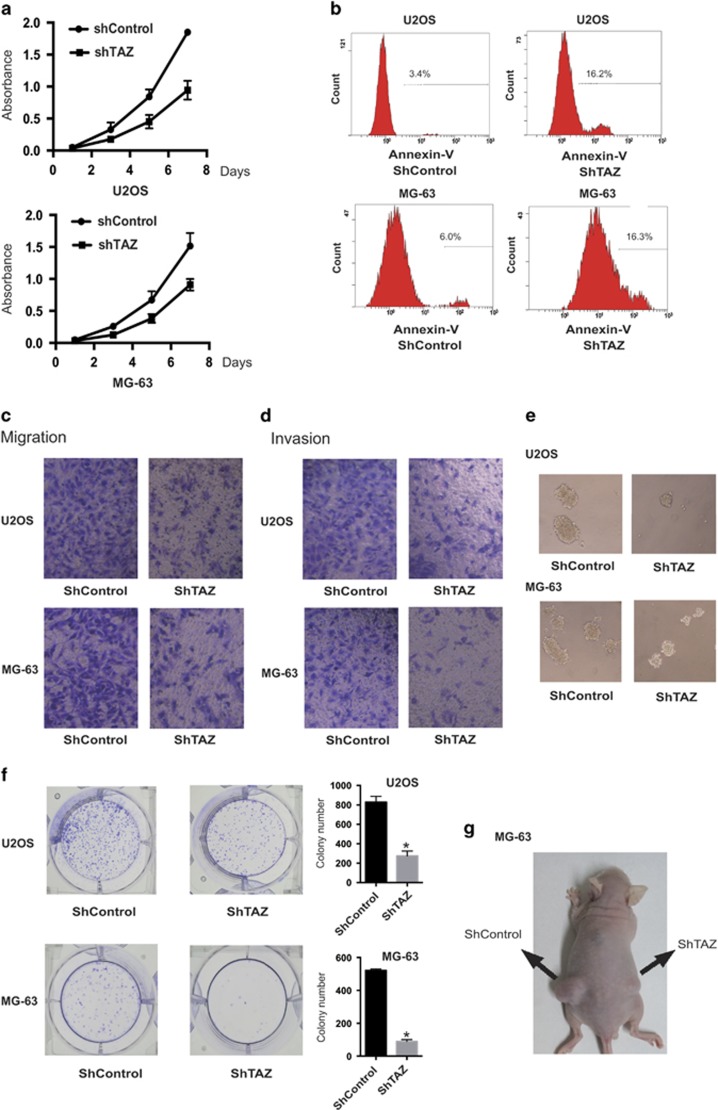
Inhibition of TAZ represses tumorigenesis. (**a**) Downregulation of TAZ expression inhibited cell proliferation in U2OS cells or MG-63 cells, compared with control cells. Absorbance was determined by the MTT assay (*n*=4, mean±S.D.). (**b**) Knockdown of TAZ promoted the apoptosis rates of U2OS cells and MG-63 cells, compared with control cells. Apoptosis rate was determined by Annexin V–PE staining and fluorescence-assisted cell sorting (FACS). (**c** and **d**) Reduced cell migration (**c**) and invasion (**d**) in U2OS cells and MG-63 cells because of TAZ knockdown. (**e**) TAZ knockdown suppressed cellular transformation. U2OS and MG-63 cells transfected with shControl or shTAZ were cultured in soft agar for 20 days and stained with crystal violet. Photographs were acquired and images were quantified with ImageJ (NIH, Bethesda, MD, USA) (details are shown in the inserts). (**f**) The same tendency was observed in cell colony formation (*n*=4, mean±S.D., *P<0.01). (**g**) Mice (*n*=3 per group) were injected with control or TAZ knockdown cells. Left and right: photographs of representative mice inoculated with MG-63 cells transfected with shRNA control or shTAZ in the left and right dorsal area, respectively

TAZ was previously demonstrated to act as an oncogene by facilitating cancer cell proliferation, invasion and transformation.^15^ To identify the function of TAZ in OS, TAZ knockdown cells of U2OS and MG-63 were generated. The rate of cell growth in both types of knockdown cells was suppressed on day 7, compared with those transfected with empty vectors (Figure 2a). Moreover, TAZ knockdown markedly enhanced OS cell apoptosis (Figure 2b).The migratory and invasive abilities of the knockdown cells were assessed by Transwell assay (Figures 2c and d). Inhibition of TAZ significantly suppressed anchorage-independent growth and colony formation, compared with controls (Figures 2e and f). To examine whether TAZ contributes to tumorigenesis, MG-63 cells transfected with the empty vector or ShTAZ were injected into nude mice (Figure 2g). Compared with the control cells, the knockdown cells exhibited compromised tumor formation. Thus, inhibition of TAZ repressed tumorigenesis both *in vitro* and *in vivo*.

### miR-224 mediates the oncogenic potential of TAZ

**Figure 3:**
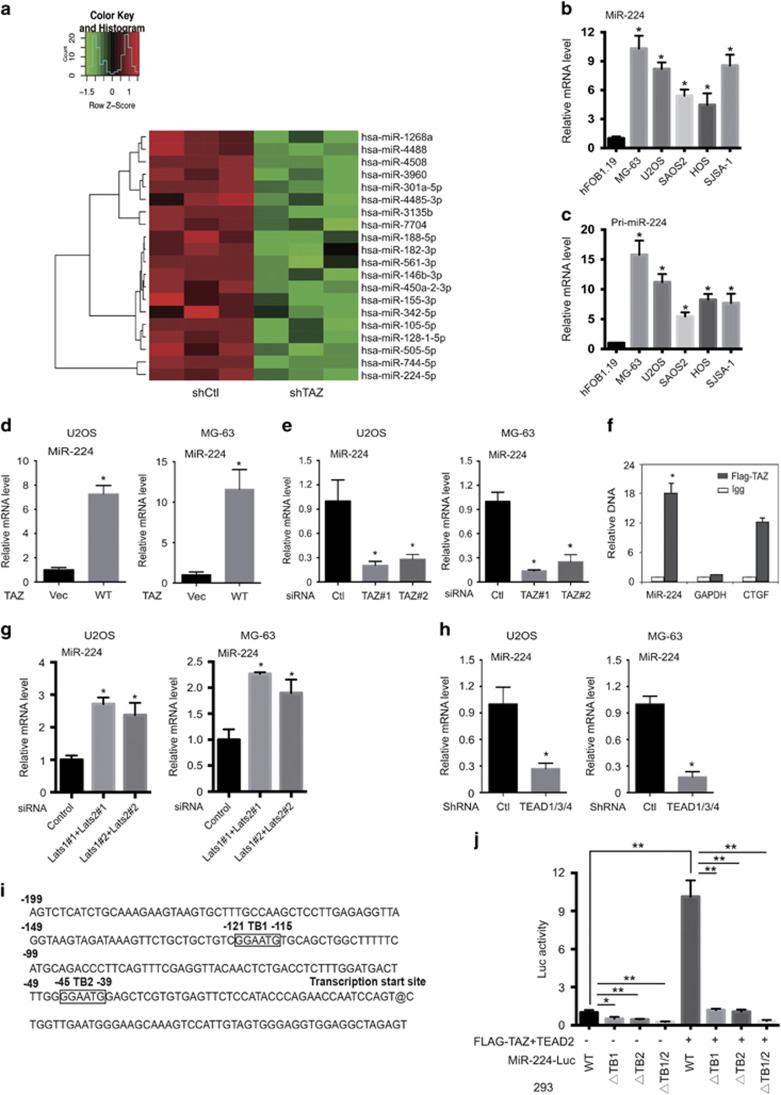
miR-224 is a direct target gene of TAZ-TEAD. (**a**) TAZ-regulated miRNAs. miRNAs regulated by TAZ were identified by miRNA high-throughput sequencing. miRNAs with a *P*-value of <0.01 in TAZ knockdown MG-63 cells were chosen, and the heat map was drawn using Matlab. (**b** and **c**) miR-224(**b**) and pri-miR-224 (**c**) were highly expressed in OS cells. Quantitative PCR (qPCR) analysis was used to quantify the endogenous levels of miR-224 and pri-miR-224 in OS cells. U6 and *β*-actin were used as controls. (**d** and **e**) TAZ induces miR-224 expression. The miR-224 expression levels in U2OS and MG-63 stable cells with TAZ overexpression (**d**) or TAZ knockdown (**e**) were determined by quantitative reverse transcription (qRT-PCR). Experiments were performed in triplicate. (**f**) TAZ binds to the promoter of miR-224. ChIP was performed using anti-FLAG or anti-IgG antibodies, followed by qPCR using primers specific to the indicated promoter regions (*n*=4, mean±S.D., *P<0.01). Data were obtained from three independent experiments. (**g**) Lats1/2 knockdown induces miR-224 expression. The miR-224 expression levels in U2OS and MG-63 stable cells with Lats1/2 knockdown were determined by qRT-PCR. Experiments were performed in triplicate. (**h**) Knockdown of TEADs reduces miR-224 level. Cells were transfected with shRNA targeting TEAD1/3/4. The levels of mature miR-224 were determined. Experiments were performed in triplicate (*n*=4, mean±S.D., *P<0.01). (**i**) The miR-224 promoter region contains two putative TEAD-binding sites. The putative TEAD-binding sites (TB1–TB2) are shown in the frame. (**j**) The putative TEAD-binding sites are important for miR-224 promoter activity. The putative TEAD-binding sites (TB) were mutated individually or in combination. The luciferase activity of each reporter was measured in the presence or absence of TAZ. The activation folds by TAZ are shown (*n*=4, mean±S.D., *P<0.01 **P<0.001)

To investigate whether miRNAs mediate TAZ functions, we profiled TAZ-induced miRNAs in MG-63 cells by high-throughput sequencing (Figure 3a). Validation of the hits revealed that miR-224 was induced by TAZ. Interestingly, the profile of the upregulated miRNAs in OS compared with those in normal tissue showed that miR-224 also acted as a potential oncogene in OS (Supplementary Table 1). Next, we validated the high expression level of miR-224 in OS cell lines by qRT-PCR (Figures 3b and c). Moreover, knockdown or overexpression of endogenous TAZ (Supplementary Figure S1c and d) substantially reduced or enhanced pri- and mature *miR-224* levels in OS cell lines (Figures 3d and e,Supplementary Figure S1a and b). These results suggested that miR-224 mediates the oncogenic potential of TAZ.

### miR-224 is a direct target of TAZ-TEAD

To determine how miR-224 mediates the function of TAZ, we investigated whether miR-224 is a direct target of TAZ and performed ChIP in TAZ overexpression stable MG-63 cells with the Flag-TAZ antibody, followed by qPCR using primers for the miR-224 promoter. The results revealed that TAZ binds to the promoter region of miR-224, particularly the region 150–300-bp upstream of the transcription start site, which contains two TEAD consensus sites (Figure 3f). *CTGF*, a known target gene of TAZ-TEAD, and *GAPDH* were used as the positive and negative controls, respectively. Hippo pathway kinases may negatively regulate miR-224 expression by inhibiting TAZ. Indeed, knockdown of Lats1/2 increased miR-224 and pri-miR-224 levels (Figure 3g, Supplementary Figures S1e and f). To check whether the induction of miR-224 by TAZ depends on TEADs, we confirmed that knockdown of TEADs reduced the expression of miR-224 (Figure 3h,Supplementary Figures S1 h–j). Furthermore, *TAZ* and TEAD2 activated the miR-224 promoter reporter in a manner dependent on the TEAD-binding site (TB1 and TB2; Figures 3i and j). Notably, the organization and position of the TB1/2 sites on the miR-224 promoter were highly similar to those on the CTGF promoter. These results indicated that miR-224 is a direct target of TAZ and TEAD.

### Overexpression of miR-224 enhances the proliferation and tumorigenicity of OS cells *in vitro* and *in vivo*

**Figure 4:**
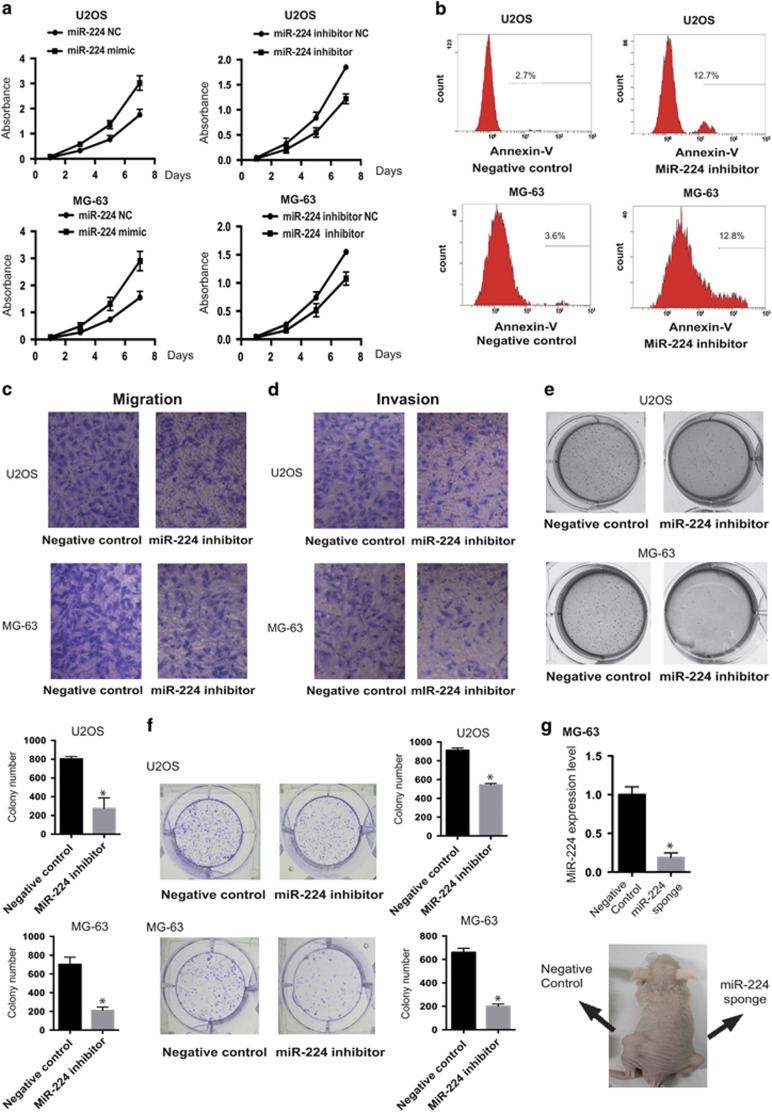
Overexpression of miR-224 enhances the proliferation and tumorigenicity of OS cells *in vitro* and *in vivo*. (**a**) Cell MTT assay. Overexpression or knockdown of miR-224 by miR-224 mimics or inhibitors affected the cell proliferation of MG-63 and U2OS cells. Data are presented as mean±S.D. (*n*=6). (**b**) Flow cytometric analysis of cell apoptosis rate. The apoptosis rate of miR-224-knockdown U2OS and MG63 cells increased significantly, compared with control cells. (**c** and **d**). Transwell migration (**c**) and invasion (**d**) assays showed that the miR-224 inhibitor suppressed the migration and invasion abilities of U2OS and MG-63 cells. (**e**) U2OS and MG-63 cells transfected with the negative control (NC) or miR-224 inhibitor were cultured in soft agar for 20 days. Colonies were stained with crystal violet, photographed and quantified with ImageJ (details are shown in the inserts). (**f**) Inhibition of miR-224 repressed cell growth, as determined by colony formation assays. Error bars represent the mean±S.D. from three independent experiments; *P<0.01. (**g**) Control or miR-224-inhibited MG-63 cells were injected into each group. Inset shows miR-224 expression in tumors from control or miR-224 sponge cells. Photograph of representative mice inoculated with the negative control or miR-224 sponge-expressing cells in the left or right dorsal area, respectively (*n*=4, mean±S.D., *P<0.01)

Given the high expression of miR-224 in both OS cells and tissues, we hypothesized that miR-224 has a role in the carcinogenesis and progression of OS. We transfected the OS cell lines, U2OS and MG-63, with hsa-miR-224 mimic or inhibitor oligonucleotides and examined the effects on cellular proliferation (Figure 4a). Expression of the *miR-224* inhibitor promoted OS cell apoptosis (Figure 4b). Besides, in response to knockdown of miR-224, the migratory and invasive abilities of the U2OS and MG-63 cells were significantly decreased (Figures 4c and d). Colony formation assays revealed that knockdown of miR-224 significantly decreased the growth rate of both the OS cell lines, compared with negative control-transfected cells (Figure 4e). Remarkably, inhibition of miR-224 also strongly suppressed anchorage-independent growth, a hallmark of oncogenic transformation. The induction of colony formation by the miR-224 inhibitor was lower in both the OS cell types than in control cells (Figure 4f).

To test whether miR-224 could promote the growth of OS tumors *in vivo*, we engineered MG-63 cells to stably knockdown miR-224. These stably miR-224 sponge and control cells were subcutaneously inoculated into nude mice. As shown in Figure 4g, the tumors in the MG-63/miR-224 sponge group grew far less rapidly than the tumors in the MG-63/vector group.

### miR-224 mediates the oncogenic potential of TAZ in OS cells *in vitro*

**Figure 5:**
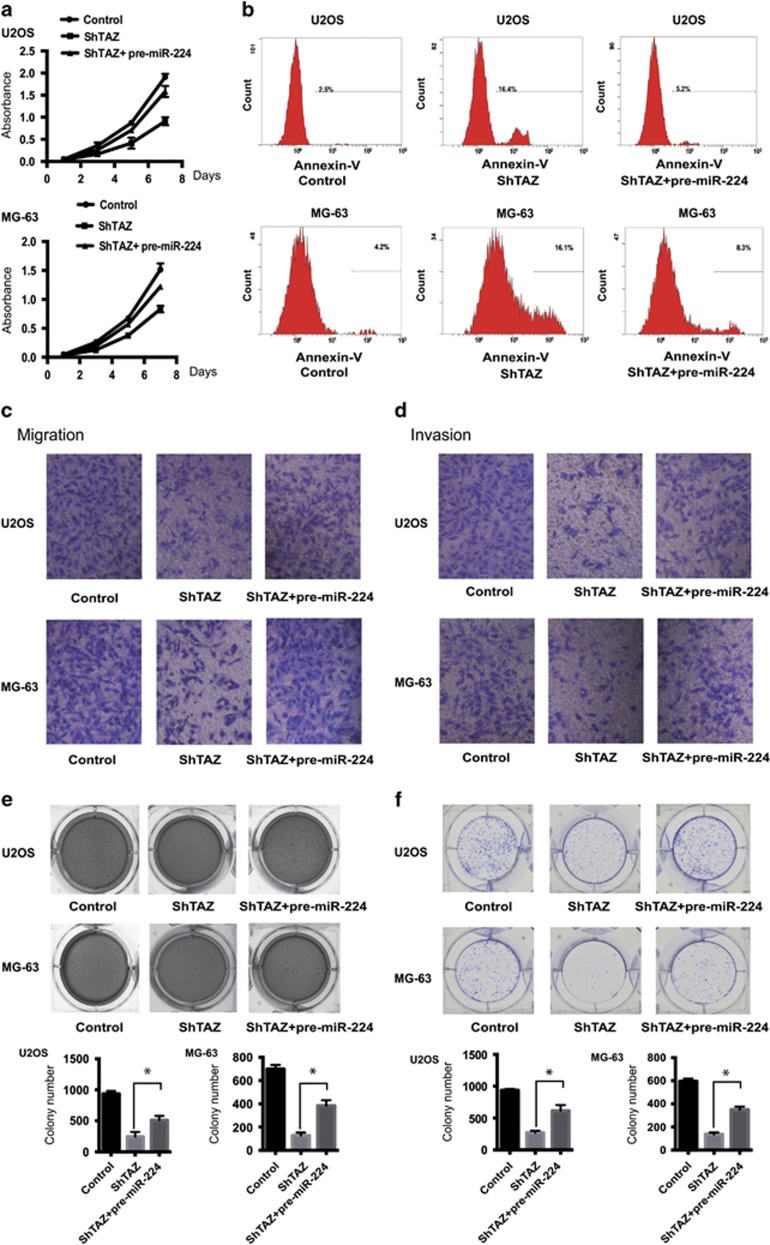
miR-224 mediates the oncogenic potential of TAZ in OS cells *in vitro*. (**a**) MTT analyses of the indicated cells, including control cells and TAZ shRNA knockdown cells transfected with the miR-224 mimic or negative control. Each bar represents the mean±S.D. of three independent experiments. (**b**) Cell apoptosis of MG-63 and U2OS cells co-transfected with pre-miR-224 and shTAZ was detected by Annexin V–PE staining. (**c** and **d**) The effect of miR-224 on migration and invasion was hampered by shTAZ. Transwell assays were performed to detect the cell migration and invasion of MG-63 and U2OS cells co-transfected with pre-miR-224 and shTAZ, compared with controls. (**e**) Soft agar colony formation ability was detected in MG-63 cells co-transfected with pre-miR-224 and shTAZ. Colonies were stained with crystal violet, photographed and quantified by using ImageJ (details are shown in the inserts; *n*=4, mean±S.D., *P<0.01). (**f**) Overexpression of miR-224 promoted the growth of TAZ knockdown cells, as determined by colony formation assays, Error bars represent the mean±S.D. from three independent experiments; *P<0.01

To elucidate whether the pro-tumorigenic function of TAZ was mediated by miR-224, we performed co-transfection studies. Interestingly, enhancement of miR-224 by the miRNA mimic hampered the growth inhibition induced by TAZ *shRNA*-treatment (Figure 5a). Consistently, expression of miR-224 cooperated with TAZ *shRNA*, and led to a decrease in the cell apoptosis rate (Figure 5b). Remarkably, overexpression of miR-224 clearly abrogated the TAZ *shRNA*-induced diminution of migration and invasion in both U2OS and MG-63 cells (Figures 5c and d). Moreover, overexpression of miR-224 also strongly rescued TAZ knockdown-induced anchorage-independent growth inhibition (Figure 5e). Besides, we investigated whether further increase of miR-224 level would promote the TAZ-induced transformation of OS cells. Indeed, a combination of TAZ shRNA and pre-miR-224 was able to promote cell colony formation (Figure 5f). These data provided further evidence that miR-224 is a TAZ target gene important for over-proliferation and tumorigenesis *in vitro*.

### miR-224 mediates the tumorigenic function of TAZ *in vivo*

**Figure 6:**
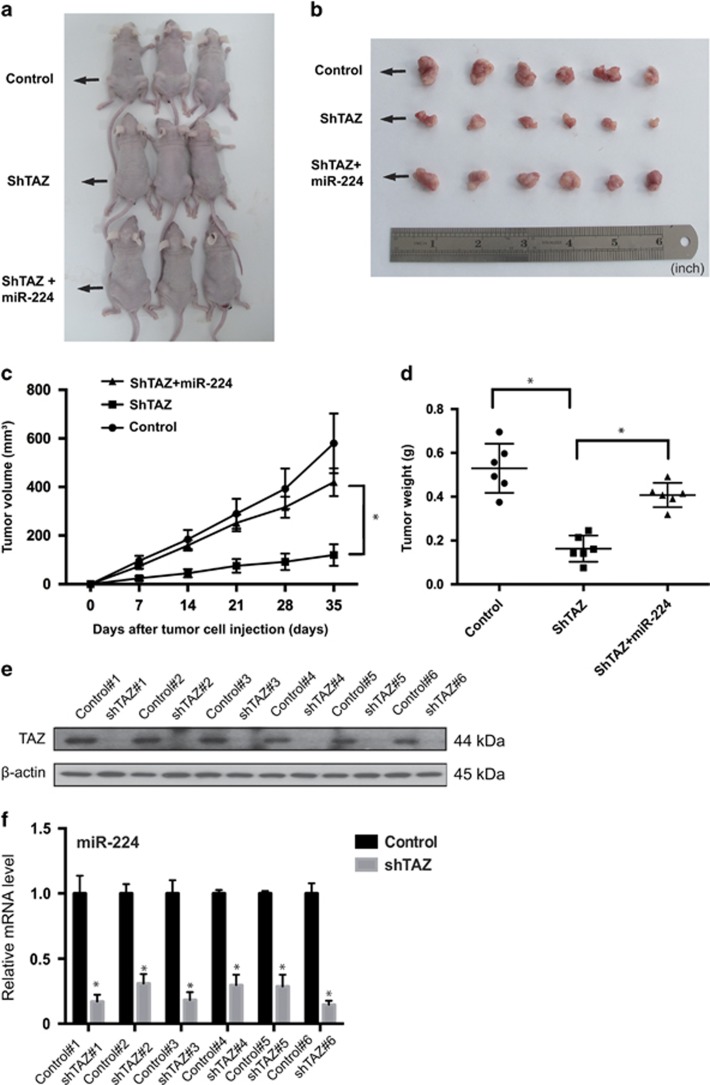
miR-224 mediates the *in vivo* tumorigenesis function of TAZ. (**a** and **b**) Nude mice were injected with 1 × 10^7^ MG-63 stable cells. Tumors were dissected and photographed after 5 weeks. (**c**) Graph represents tumor volumes at the indicated days for the three groups: control, shTAZ and co-transfection of shTAZ and pre-miR-224; six mice in each group. Data are presented as mean±S.D. (*n*=6), *P<0.01. (**d**) Tumor weight averages of each mice group at the end of the experiment (day 35). Data are presented as mean±S.D. (*n*=6), *P<0.01. (**e**) Immunoblot analysis of TAZ in tumors from xenograft mice. (**f**) qRT-PCR analysis of miR-224 in tumors from xenograft mice

To examine the *in vivo* pro-tumor growth effects of miR-224 in OS, MG-63 cell lines with TAZ knockdown, or transfected with pre-miR-224 and control vector, were subcutaneously injected into nude mice, and tumor growth was evaluated. MG-63 cells with TAZ knockdown and miR-224 upregulation exhibited a significantly higher growth rate than that of TAZ-knockdown cells (Figures 6a and b). Inversely, knockdown of TAZ in MG-63 cells caused a substantial reduction in tumor volume *in vivo* (Figure 6c), whereas co-expression of shTAZ and pre-miR-224 rescued the decrease in tumor volume. In addition, the differences in tumor average wet weight among the three groups were similar to the differences in tumor volume (Figure 6d). The knockdown expression efficiency of TAZ is illustrated in Figure 6e. Thus, miR-224 is a TAZ target gene and is important for tumorigenesis *in vivo*.

### SMAD4 is a target of miR-224 in OS

**Figure 7:**
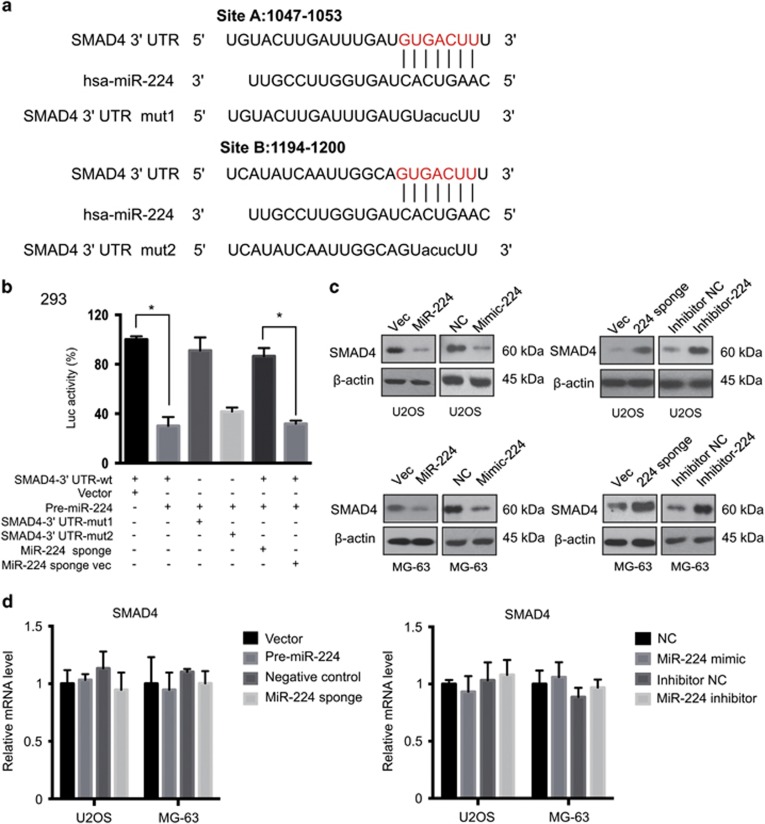
SMAD4 is a direct target of miR-224. (**a**) Schematic diagram showing the matched sequences between the seed sequence of miR-224 and the 3′-UTRs of SMAD4. The lower case alphabets indicate the mutated nucleotides. (**b**) Luciferase reporter constructs containing WT or mutated SMAD4 3′-UTRs were co-transfected with pre-miR-224 or miR-224 sponge into 293T cells (*n*=4, mean±S.D., *P<0.01). (**c**) miR-224 represses SMAD4 protein level. Cells were transfected with NC or miR-224 mimic or infected with empty vector or pre-miR-224. Cell lysates were subjected to immunoblotting. miR-224 inhibition increases SMAD4 protein level. Cells were transfected with NC or miR-224 inhibitor or infected with empty vector or miR-224 sponge, and analyzed for SMAD4 protein level. (**d**) Knockdown or overexpression of miR-224 does not influence SMAD4 mRNA level. Total RNA was extracted from U2OS and MG-63 cells transfected with the indicated plasmids, and SMAD4 mRNA levels were determined by qRT-PCR (*n*=4, mean±S.D.)

To dissect how miR-224 functions downstream of TAZ, we used the PicTar and TargetScan algorithms to predict potential miR-224 targets. Among these targets, the 3′-untranslated regions (UTRs) of SMAD4 mRNAs contained sequences complementary to the miR-224 seed sequence (Figure 7a). We focused on *SMAD4* because it has a tumor-suppressive role in carcinogenesis. To verify whether *SMAD4* is a direct target of miR-224, we constructed a *SMAD4* 3′-UTR sensor and co-transfected the 3′-UTR and pre-miR-224 in 293T cells. Consistent reduction in luciferase activity for *SMAD4* 3′-UTR by miR-224 was observed (Figure 7b). To validate target specificity, we generated mutated forms of the 3′-UTR, where the binding sites of miR-224 were destroyed using the QuikChange Mutagenesis Kit. Briefly, there are two miR-224-binding sites on the 3′-UTR of SMAD4. Co-transfection of pre-miR-224 with the mutated forms of the 3′-UTRs significantly attenuated the reduction of luciferase activities on wild-type (WT) 3′-UTR (Figure 7b), indicating specific binding of miRNA and target SMAD4 3′-UTR. Furthermore, transfection of the miR-224 mimic or expression of pre-miR-224 substantially repressed endogenous SMAD4 protein level (Figure 7c, Supplementary Figure S2a). Moreover, inhibition of endogenous miR-224 by an inhibitor antisense oligomer or miRNA sponge clearly increased SMAD4 protein levels in both cell lines (Figure 7c, Supplementary Figure S2b). However, miR-224 did not significantly alter the SMAD4 mRNA level (Figure 7d), suggesting the presence of an underlying mechanism for translation repression. Together, these results suggest that SMAD4 is a miR-224 target that is critical for growth regulation.

### SMAD4 has essential roles in the manifestation of miR-224–induced phenotypes

**Figure 8:**
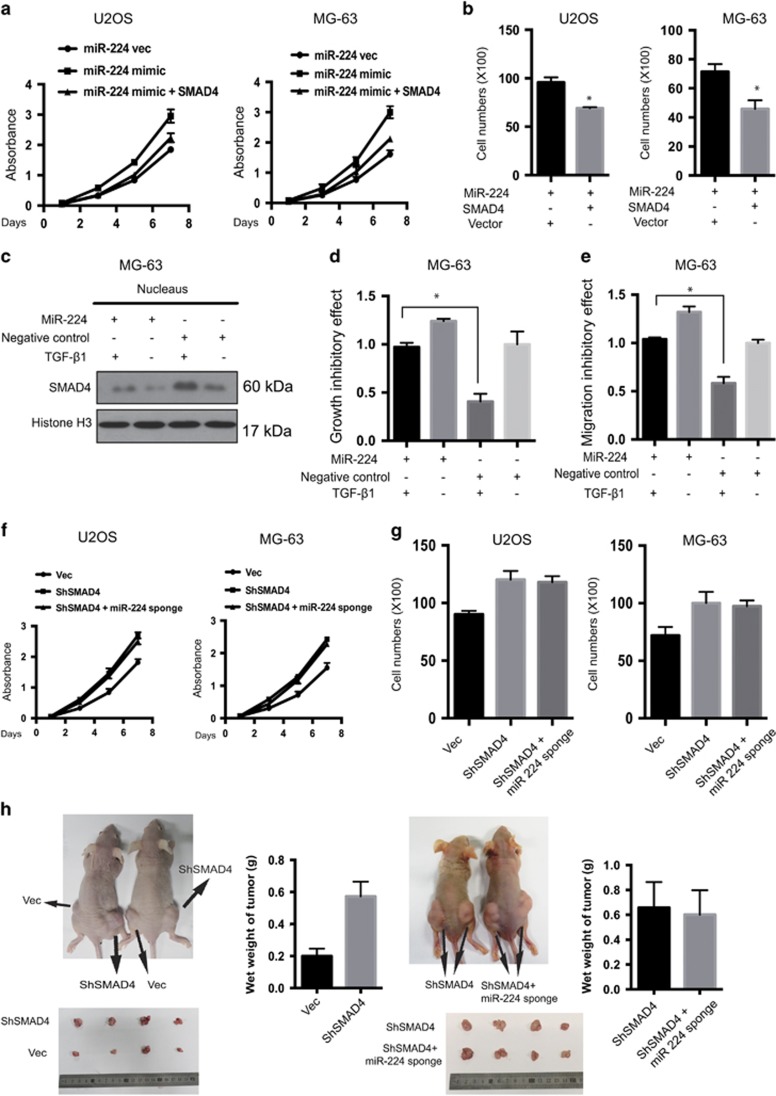
SMAD4 has a crucial role in miR-224–induced phenotypes. (**a**) Cell proliferation assay for SMAD4 and miR-224 overexpression in U2OS and MG-63 OS cells. The cell growth rates were measured by the MTT assay (*n*=4, mean±S.D.). (**b**) SMAD4 reduced the migration ability induced by miR-224 overexpression. Cell migration assay of miR-224-overexpressing cells transfected with SMAD4 or empty vector (*n*=4, mean±S.D., *P<0.01). (**c**) pre-miR-224 and MG-63 NC cells were treated with or without TGF-*β* for 8 h. Nuclear proteins were extracted and subjected to western blot analysis. (**d** and **e**) pre-miR-224 and MG-63 NC cells were treated with or without TGF-*β* for 8 h. The growth inhibitory effect and migration ability were measured by (**d**) cell proliferation assay and (**e**) migration assay. The values represent the means±S.D., *P<0.01. (**f**) Cell proliferation assay for SMAD4 and miR-224 knockdown in U2OS and MG-63 OS cells. The cell growth rates were measured by the MTT assay (*n*=4, mean±S.D.). (**g**) MiR-224 knockdown had no effect on the migration inhibition ability induced by SMAD4 knockdown. Cell migration assay of SMAD4 knockdown cells transfected with miR-224 sponge or control vector (*n*=4, mean±S.D., *P<0.01). (**h**) Nude mice were injected with 1 × 10^7^ MG-63 stable cells. Tumors were dissected and photographed after 5 weeks

Given the functional importance of SMAD4 on miR-224-mediated cell growth and migration in OS cells, we co-transfected SMAD4 and the miR-224 mimic into U2OS and MG-63 cells. miR-224-induced cell proliferation and migration was partially blocked by SMAD4 overexpression (Figures 8a and b,Supplementary Figures S3a and b) in both U2OS and MG-63 OS cells. SMAD4 has an essential role in the TGF-*β* signaling pathway, where it binds to SMAD2/3 and translocates to the nucleus upon TGF-*β* stimulation.^30^ To evaluate whether miR-224 affects TGF-*β* signaling by targeting SMAD4, we further treated miR-224-overexpressing and control cells with TGF-*β*. miR-224 impaired the TGF-*β*–induced nuclear transportation of SMAD4 to some extent (Figure 8c), and accordingly, miR-224 markedly attenuated the TGF-*β-*induced restrain of cell growth and migration in MG-63 cells (Figures 8d and e). These results suggest that miR-224 has a momentous role in the TGF-*β* signaling pathway by targeting SMAD4 in OS. Furthermore, we generated cells with stable knockdown of SMAD4, and further transfected them with miR-224 sponge. miR-224 knockdown had no impact on SMAD4 knockdown-induced cell proliferation (Figure 8f), migration (Figure 8g) or tumorigenesis (Figure 8h). Together, these results suggest that SMAD4 has essential roles in the manifestation of miR-224-induced phenotypes.

### SMAD4 has essential roles in the manifestation of TAZ-induced phenotypes

**Figure 9:**
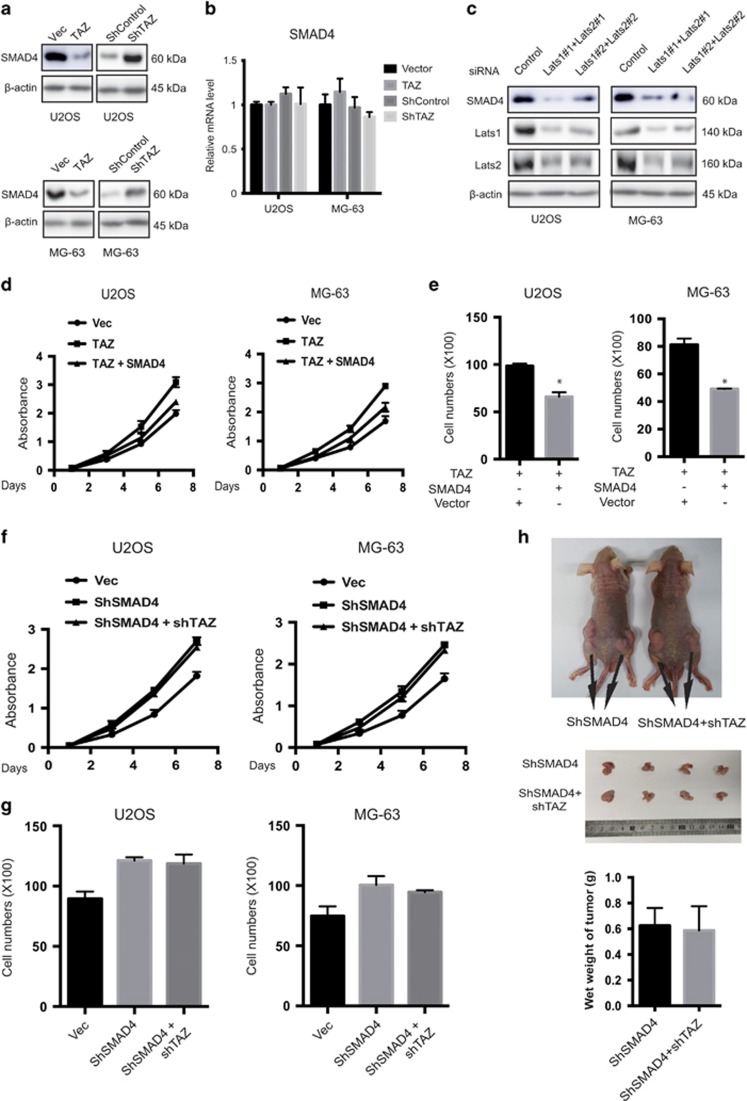
SMAD4 has a crucial role in TAZ-induced phenotypes. (**a**) TAZ represses SMAD4 protein level. Cells were infected with empty vector or flag-TAZ, and shRNA control or shTAZ. Cell lysates were subjected to immunoblotting. (**b**) Knockdown or overexpression of TAZ does not influence SMAD4 mRNA level. Total RNA was extracted from U2OS and MG-63 cells transfected with the indicated plasmids, and SMAD4 mRNA levels were determined by qRT-PCR (*n*=4, mean±S.D.). (**c**) Knockdown of Lats1/2 represses SMAD4 protein level. Cells were transfected with siRNAs as indicated and analyzed by immunoblotting. (**d**) Cell proliferation assay for SMAD4 and TAZ overexpression in U2OS and MG-63 OS cells. The cell growth rates were measured by the MTT assay (*n*=4, mean±S.D.). (**e**) SMAD4 reduced the migration ability induced by TAZ overexpression. Cell migration assay of TAZ-overexpressing cells transfected with SMAD4 or empty vector (*n*=4, mean±S.D., *P<0.01). (**f**) Cell proliferation assay for SMAD4 and TAZ knockdown in U2OS and MG-63 OS cells. The cell growth rates were measured by the MTT assay (*n*=4, mean±S.D.). (**g**) TAZ knockdown had no effect on the migration inhibition ability induced by SMAD4 knockdown. Cell migration assay of SMAD4 knockdown cells transfected with shTAZ or shControl vector (*n*=4, mean±S.D., *P<0.01). (**h**) Nude mice were injected with 1 × 10^7^ MG-63 stable cells. Tumors were dissected and photographed after 5 weeks

Given the functional importance of miR-244 on TAZ-mediated cell growth and migration in OS cells, we next sought to elucidate the function of SMAD4 in TAZ-induced phenotypes. Similar to miR-224, expression of TAZ substantially repressed endogenous SMAD4 protein levels (Figure 9a). Moreover, inhibition of endogenous TAZ by shRNA clearly increased SMAD4 protein levels in both cell lines (Figure 9a). However, TAZ did not significantly alter SMAD4 mRNA levels (Figure 9b). Through inhibition of TAZ, the Hippo pathway kinases may function as positive regulators of SMAD4 protein expression. Indeed, knockdown of Lats1/2 decreased SMAD4 protein levels (Figure 9c).

Next, we co-transfected SMAD4 and TAZ into U2OS and MG-63 cells. TAZ-induced cell proliferation and migration was partially blocked by SMAD4 overexpression (Figures 9d and e) in both U2OS and MG-63 OS cells. Further, we generated cells with stable knockdown of SMAD4, and then co-transfected them with TAZ shRNA. TAZ knockdown had no impact on SMAD4 knockdown-induced cell proliferation (Figure 9f), migration (Figure 9g) or tumorigenesis (Figure 9h). In conclusion, these results suggest that SMAD4 has essential roles in the manifestation of TAZ-induced phenotypes.

### TAZ and miR-224 affect TGF-B and BMP signaling

**Figure 10:**
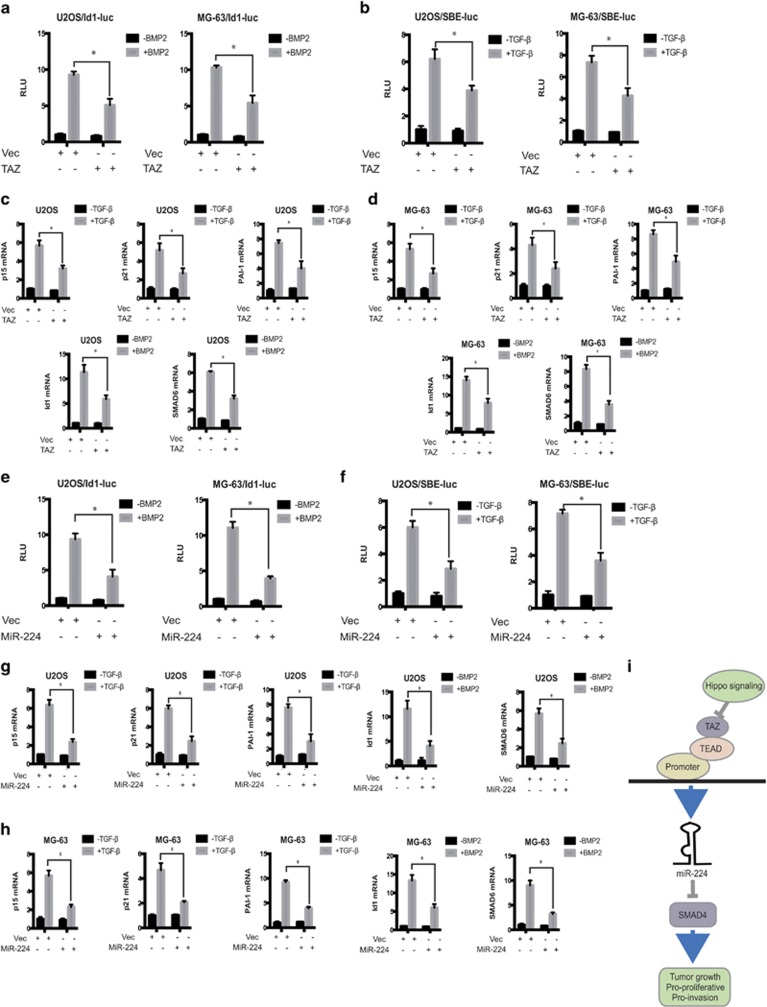
TAZ and miR-224 affect TGF-B and BMP signaling. (**a**) TAZ inhibits bone morphogenetic protein (BMP) 2-induced Id1-luc reporter activity. U2OS and MG-63 cells were transfected with vector or flag-TAZ, together with Id1-luc reporter plasmids. BMP2 treatment and luciferase assays were performed as described under 'Materials and methods' section. (**b**) TAZ inhibits TGF-*β*-induced SBE-luc reporter activity. U2OS and MG-63 cells were transfected with vector or flag-TAZ, together with SBE-luc reporter plasmid, and then treated with TGF-*β* for 12 h. (**c** and **d**) Stable expression of TAZ in U2OS (**c**) and MG-63 (**d**) cells decreases mRNA levels of p15, p21, PAI-1, Id1 and SMAD6. Cells were treated with or without TGF-*β* or BMP2 for 12 h, and total RNA was extracted for qRT-PCR analysis. (**e**) MiR-224 inhibits BMP2-induced Id1-luc reporter activity. U2OS and MG-63 cells were transfected with vector or pre-miR-224, together with Id1-luc reporter plasmids. BMP2 treatment and luciferase assays were done as described under 'Materials and methods' section. (**f**) MiR-224 inhibits TGF-*β*-induced SBE-luc reporter activity. U2OS and MG-63 cells were transfected with vector or pre-miR-224, together with SBE-luc reporter plasmid, and then treated with TGF-*β* for 12 h. (**g** and **h**) Stable expression of miR-224 in U2OS (**g**) and MG-63 (**h**) cells decreases mRNA levels of p15, p21, PAI-1, Id1 and SMAD6. Cells were treated with or without TGF-*β* or BMP2 for 12 h, and total RNA was extracted for qRT-PCR analysis. (**i**) Schematic model of the potential signaling mechanism of the TAZ–miR-224–SMAD4 axis

We investigated the effects of TAZ and miR-224 overexpression on BMP-induced transcriptional responses using reporter assays. The U2OS and MG-63 cell lines are responsive to BMP. Overexpression of TAZ and miR-224 significantly inhibited BMP-induced transcriptional activation of the natural Id1 promoter (Figures 10a and e), as well as TGF-*β*-induced transcriptional activation (Figures 10b and f) in both the cell lines.

To further characterize the effect of TAZ and miR-224 overexpression on the expression of endogenous TGF-*β* target genes, we examined the levels of p15Ink4b and p21Cip1 mRNAs in cells overexpressing TAZ and miR-224. Although TGF-*β* upregulated the mRNA levels of both p15Ink4b and p21Cip1, overexpression of TAZ (Figures 10c and d) and miR-224 (Figures 10g and h) decreased these levels in U2OS and MG-63 cells. Similarly, PAI-1 mRNA levels were upregulated and diminished in response to TGF-*β* treatment and overexpression of TAZ (Figures 10c and d) and miR-224 (Figures 10g and h), respectively.

We next investigated whether overexpression of TAZ and miR-224 potentiates BMP responses. Notably, overexpression of TAZ (Figures 10c and d) and miR-224 (Figures 10g and h) markedly decreased the expression of BMP downstream genes such as Id1 and SMAD6 in U2OS and MG-63 cells. Together, these data strongly suggested that TAZ and miR-224 are both regulators of TGF-B and BMP signaling.

## Discussion

The Hippo pathway has a dominant role in organ size control, growth and development, and tissue homeostasis. Recently, increasing evidences have revealed the close relationship of the Hippo pathway with malignant tumors. As a regulator and vital molecule in the upstream reactions of the tumor-suppressor pathway, mutation or deletion of the Hippo pathway upregulates YAP/TAZ expression, which promotes tumorigenesis and influences prognosis.^31, 32^ YAP regulates the corresponding procedures by controlling miRNAs. However, few studies have demonstrated the regulation of miRNAs through TAZ. The Hippo pathway is highly complex and variable, with multiple cross-talks with other signaling pathways.^33^ Notably, miR-29 has been identified as a YAP target gene mediating cross talk with the mTOR pathway and affecting cell size.^34^ These results suggested that the mammalian Hippo pathway could function via miRNA targets. Moreover, recently, a functional miR-130a mediating the organ size control activity of YAP *in vivo* was identified.^6^ TAZ is a homolog of YAP that contributes to preserving the pluripotency of breast cancer,^35, 36^ and the results of this study suggest that it is overexpressed in OS tissues and cells. However, few researchers have reported that miRNA mediates TAZ function and that TAZ regulates miRNA. In this study, cell lines with upregulated TAZ expression were chosen to study the regulation of miRNA by TAZ. We found TAZ-regulated miR-224 and TAZ were both associated with the TGF-*β* pathway. Our results showed that the regulatory network of miR-224 has a significant effect on proliferation, invasion and migration.

The TGF-*β* and Hippo pathways exhibit cross-talk in the tumorigenesis of OS.^37^ The downstream molecules of the TGF-*β* and Hippo pathways function as transcriptional regulators, controlling the transcription of target genes. In a previous study, YAP/TAZ restricted the accumulation and transcriptional activity of SMAD when an aberrant Hippo pathway was activated.^38^ In dense cells, the Hippo pathway was activated, and YAP/TAZ was restrained within the cells. Then, SMAD attenuated TGF-*β* transcription, which in turn suppressed epithelial-to-mesenchymal transition (EMT).^39^ The Hippo pathway influences SMAD function through miRNA, and has a vital effect on stem cells, cancer stem cells, and strong proliferative cells. Previously, we believed that YAP/TAZ possessed dual function, including the promotion of proliferation by upregulating CTGF and BIRC5 and inhibition of apoptosis by downregulating the corresponding genes. For instance, overexpression of YAP/TAZ or aberrant activation of YAP/TAZ caused by mutations of other factors in the Hippo pathway could facilitate the expression of downstream transcription factors like cyclin E and DIAP1, which led to the inhibition of apoptosis.^40^ Our model described and complemented the anti-apoptotic function of TAZ and enriched the variability of the network involving TGF-*β*, miRNA and Hippo.

The TGF-*β* signaling pathway induces EMT during cancer progression. A recent report revealed that CTGF is required for TGF-*β*-induced EMT.^41^ Although TAZ is thought to contribute to EMT,^42^ the molecular mechanism by which the transcription factor achieves its deleterious effects is unknown. Although we identified the cross talk of the two pathways and demonstrated that miR-224-mediated migration is controlled by TAZ, the specific mechanisms of invasion and migration need to be clarified in future studies.

In conclusion, in this study, we demonstrated that the expression levels of TAZ and miR-224 are upregulated in OS tissues. Moreover, we proposed a novel TAZ–miR-224–SMAD4 axis in OS cells that mediates cell invasion and migration, and drives cell proliferation and tumor growth. These findings suggest that TAZ and miR-224 may represent new targets for OS therapy. Notably, the similarity of YAP and TAZ in TEAD-binding indicates that YAP may mediate miR-224 onco-activity in a similar manner, although we did not investigate the role of miR-224 in mediating YAP function in this study. Technical advances on antisense miRNA oligomers (antimiRs) have rendered miRNAs drug targets for anticancer therapy with unique advantages.^43^ Thus, the identification of the remarkable role of miR-224 in sustaining TAZ activity would pave the way for modulating TAZ-miR-224 and TGF-*β* as exciting new therapeutic targets for OS with high TAZ expression.

## Materials and methods

### Ethics

All animal experiments were carried out according to relevant national and international guidelines and approved by the Stanford Institutional Animal Care and Use Committee (IACUC). All experiments strictly followed the panel's specific guidelines regarding the care, treatment and killing of animals used in the study.

### Clinical samples

Slices of formalin-fixed and paraffin-embedded primary OS and chondroma tissues were obtained from biopsies in 16 and 20 patients before administration of neo-adjuvant chemotherapy according to the Chinese national ethical guidelines (‘Code for Proper Secondary Use of Human Tissue', Chinese Federation of Medical Scientific Societies). Adjacent normal bone tissue samples were obtained from these 16 OS patients after surgical resection. OS, chondroma and normal bone tissue biopsies were histologically characterized by pathologists according to the criteria defined by the World Health Organization. Written informed consent was obtained from each patient before entering this study, and all study protocols were approved by the Ethics Committee for Clinical Research of Zhejiang University, Hangzhou, China.

### Immunohistochemistry staining

Immunohistochemical analysis was performed. The primary antibodies against TAZ was diluted 1 : 200 and were incubated at 4 °C overnight in a humidified container. After washing with PBS three times, the tissue slides were treated with a non-biotin horseradish peroxidase detection system according to the manufacturer's instructions (Dako, Copenhagen, Denmark).

### Antibodies, plasmids, mice and other materials

Information regarding the materials used in this study is included in the Supplementary Materials and Methods.

### Cell culture, transfection and viral infection

Cell culture procedures are described in the Supplementary Materials and Methods.

### Xenograft tumorigenesis model

Nude mice (nu/nu, male 3- to 4-week-old) were injected subcutaneously with 1 × 10^7^ MG-63 stable cells. Tumor volumes were calculated from the length (a) and the width (b) by using the following formula: volume (ml^3^)=ab^2^/2. Five weeks after injection, the animals were killed, and tumors were harvested (measured and weighed) and fixed in 4% paraformaldehyde. Wet tumor weight was calculated as mean weight±S.D. in each group.

### Luciferase assays

For miR-224 promoter reporter and 3′-UTR reporter luciferase assays, HEK293T cells were transfected with the reporter, together with CMV-*β*-gal and the indicated plasmids. Twenty-four hours after transfection, cells were lysed. Luciferase activity was measured by using the Luciferase Assay System (Promega, Madison, WI, USA) following the manufacturer's instructions. All luciferase activities were normalized to *β*-gal activity.

For TGF-*β* and BMP luciferase reporter assays, Id1-luc and SBE-OC-luc were used to measure BMP-induced transcription, and SBE-luc for TGF-*β*-induced transcription. Cells were transfected with reporter plasmids together with Renilla luciferase plasmid to normalize transfection efficiency. Briefly, 24 h after transfection, cells were treated with BMP2 (20 ng/ml) or TGF*-β* (2 ng/ml) for 12 h. Cells were then harvested, and luciferase activity was measured using Dual-Luciferase Reporter Assay System (Promega). All assays were carried out in triplicate and normalized against Renilla Luciferase activity.

### Chromatin immunoprecipitation (ChIP)

ChIP assay was performed using the Millipore ChIP kit (Millipore, Billerica, MA, USA) according to the manufacturer's instructions. Briefly, cells were cross-linked and lysed, and DNA fragments were sonicated to an average size of 0.5 kb. ChIP was then performed using 5 *μ*g antibodies against Flag or control IgG, after 14 h of incubation. The immunoprecipitants were washed and eluted. The eluents were then de-cross-linked, and DNA was purified for polymerase chain reaction (PCR) analysis.

### RNA isolation and real-time PCR

To determine the mRNA expression levels of regular genes and pri-miR-224, total RNA was isolated from cultured cells using TRIzol reagent (Life Technologies, Carlsbad, CA, USA). cDNA was synthesized by reverse transcription using random hexamers and subjected to real-time PCR with the indicated primers in the presence of SYBR Green (Applied Biosystems, Thermo Fisher Scientific, Waltham, MA, USA). *β*-Actin was used as a standard for normalization. miRNAs were extracted using the mirVanamiRNA isolation kit (Life Technologies). The relative expression levels of the miRNAs were determined using TaqmanmiRNA Assays (Life Technologies) normalized to RNU6B. All experiments were performed at least in triplicate. Detailed experimental procedures can be found in the Supplementary Materials and Methods.

### Migration and invasion assays

Invasion assays were performed using a 24-well invasion chamber system (Corning Inc., New York, NY, USA). Cells were trypsinized and counted with a hemocytometer using Trypan blue, and viable cells were seeded in the upper chamber at a density of 3 × 10^4^ cells per well in serum-free medium. The lower chamber received 500 *μ*l of 10% fetal bovine serum-containing medium. Incubation was carried out for 36 h at 37 °C in humidified air with 5% CO_2_. Migrated cells on the lower surface were fixed with methanol and stained with Giemsa. The number of migrating cells was determined by counting five high-powered fields (200 ×) on each membrane. The migration assay was a modification of the invasion assay described previously. A total of 3 × 10^4^ cells were placed in the upper chamber in serum-free medium. The lower chamber was filled with medium containing 10% fetal bovine serum. Cells were allowed to migrate through a porous, uncoated membrane for 24 h at 37 °C. The membrane was processed as described for the invasion assay. The number of migrating cells was determined by counting five high-powered fields (200 ×) on each membrane. Each test group was assayed in triplicate.

### MTT assay, colony formation assay, soft agar colony formation assay, apoptosis flow cytometry and immunohistochemistry

The MTT assay, colony formation assay, soft agar colony formation assay, apoptosis flow cytometry and immunohistochemistry (IHC) were conducted according to previously described methods.^44^ Further details are provided in the Supplementary Materials and Methods.

### Statistical analysis

Statistical analyses were performed with SPSS (SPSS Inc, Chicago, IL, USA). Data are represented as means with S.D., and statistical significance was determined with unpaired Student's *t*-tests, unless indicated otherwise. *P*-values <0.05 were considered statistically significant.
