# Supplementary material for: The TAZ–miR-224–SMAD4 axis promotes tumorigenesis in osteosarcoma
Source: Cell Death Dis. 2017 Jan 5;8(1):e2539–. doi: 10.1038/cddis.2016.468 (PMC5386375; doi:10.1038/cddis.2016.468)
Supplement: Supplementary Table 1 [file cddis2016468x1.docx]

| miR-name | Ratio of normalized copy number |
| --- | --- |
|  | Osteosarcoma(OC) /normal(N) (average) |
| miR-31 | 45.2694 |
| miR-210 | 23.7583 |
| miR-767-5p | 13.7639 |
| miR-744 | 12.3374 |
| miR-148a | 10.5326 |
| miR-3178 | 10.2693 |
| miR-105 | 9.8750 |
| miR-224 | 8.7345 |
| miR-193a-5p | 8.2936 |
| miR-3188 | 7.3547 |

Supplementary Table1 1. The relative copy number of miRNAs
