# Supplementary material for: The TAZ–miR-224–SMAD4 axis promotes tumorigenesis in osteosarcoma
Source: Cell Death Dis. 2017 Jan 5;8(1):e2539–. doi: 10.1038/cddis.2016.468 (PMC5386375; doi:10.1038/cddis.2016.468)
Supplement: Supplementary Materials and Methods [file cddis2016468x2.docx]

**Supplementary Materials and Methods**

***Antibodies, plasmids, mice and other materials***

Anti-TAZ antibodies were from BD Biosciences. Anti-SMAD4 antibodies were obtained from Abcam. Anti-β-actin and anti-Flag antibodies were obtained from Sigma. Alexa Fluor 488- and Alexa Fluor 594- conjugated secondary antibodies were obtained from Life Technologies. Horseradish peroxidase-conjugated secondary antibodies were obtained from GE Healthcare. The pLKO1-shTAZ and shTEAD1/3/4 constructs were described before. TAZ wt was also subcloned into the pLVX-Flag lentiviral vector. miR-224 promoter reporter was made by cloning the -1280-+80 region of miR-224 promoter into the pGL4.21 vector. Potential TEAD binding sites were then mutated by site-directed mutagenesis. SMAD4 3’UTR sensor was made by cloning a 1660 bp fragment of SMAD4 3’UTR containing the conserved miR-224 binding site into the pMIR-REPORT vector. The miR-130a binding site was then mutated by site-directed mutagenesis. Pre-miR-224 precursor (pre-miR-224) was purchased from Ambion (Foster City, CA, USA). miR-224 sponge was made by cloning a stretch of three perfect match miR-224 antisense oligomer into the LV3 (H1/GFP&Puro) lentiviral vector. SiRNA oligonucleotides toward human TAZ and control siRNA toward luciferase were purchased from Dharmacon or RiboBio. miR- 224 mimics/ inhibitors were obtained from Shanghai GenePharma. Common chemicals were from Sigma or Sangon Biotech.

***Cell culture, transfection, and viral infection***

human fetal osteoblasts hFOB1.19 was cultured in DMEM-F12 medium (Invitrogen, Karlsruhe, Germany) and Human osteosarcoma cell lines MG-63, HOS, SJSA-1, SAOS2, U2OS were cultured in RPMI-1640 medium (Invitrogen, Karlsruhe, Germany) supplemented with 10% v/v heat-inactivated fetal bovine serum (Invitrogen) and 1% v/v penicillin/streptomycin (Invitrogen), under adherent conditions at 37 °C in a humidified incubator with 5% CO 2 /95% air.

Transfection of plasmids was performed using Lipofectamine (Life Technologies) according to the manufacturer’s instructions. Transfection of microRNA mimics/inhibitors or siRNAs was performed using Lipofectamine RNAiMAX (Life Technologies) according to the manufacturer’s instructions.

Lentiviral infection was used to generate various stable cells. Briefly, HEK293T cells were co-transfected with packaging plasmids, as well as viral vectors. 48 hours after transfection, culture medium was supplemented with 5μg/ml polybrene, filtered through a 0.45μm filter, providing for target cells. 36 hours after infection, cells were selected with 2μg/ml puromycin in culture medium.

***MTT assay for cell growth***

For the MTT assay, the cells were seeded in 96-well plates at 2×103 cells/well and incubated for 1, 3, 5 and 7 days. At each time point, MTT (Sigma-Aldrich) was added into each well. After 4 h of incubation, the resulting formazan was then dissolved in 100 μl of dimethyl sulfoxide (DMSO; Sigma-Aldrich), and the absorbance was determined at 570 nm using Versamax microplate reader (Molecular Devices, CA, USA).

***Colony formation assay***

Approximately 1 × 10 3 cells of respective treated MG63 or U2OS cells were plated in 10 cm culture dishes. Cells were fixed with methanol and stained with 0.1% crystal violet after 14 days. Clones containing over 50 cells were counted manually. The experiments were repeated three times to obtain the average colony formation rate.

***Soft agar colony formation assay***

About 2.5×104 cells were mixed with 3 ml growth medium containing 0.4% agarose and layered onto 2 ml of 0.75% agarose/ medium beds in 6-well plates. Cells were fed with 2 ml growth medium every 3 days for 3 weeks, after which colonies were stained and counted.

***Cell apoptosis analysis with flow cytometry***

MG-63 and U2OS Cells for detection were harvested and apoptosis was analyzed by flow cytomety, using Annexin V – fluorescein isothiocyanate (FITC) apoptosis detection kit (Invitrogen, Carlsbad, CA, USA), according to the manufacturer’s protocol. Data were collected on a BD FACSCanto.

***Immunohistochemistry***

Surgically resected osteosarcoma and osteochondromas were fixed in neutral buffered formalin for 24 hours at 4°C then embedded and processed according to standard protocols. The sections were deparaffinized through graded ethanol solutions. After an antigen retrieval procedure of 30 min using target retrieval solution (DAKO), the sections were stained with specific antibodies using the avidin-biotin complex system (Vector Laboratory). 3, 3'- diaminobenzidine (DAB) was used as substrate. Cell nuclei were counterstained with Hematoxylin.

***Western blotting***

Western blotting was performed according to protocol. Briefly, cells were first lysed in the protein Lysis Buffer, subjected to SDS-PAGE and then transferred to NC membranes. The membranes were blocked with 5% Nonfat milk and then incubated with antibodies, then washed with TBST. Protein expression was detected by chemiluminescence (ECL, Amersham, Piscataway, NJ). The expression of β-actin was used as a loading control.

***MicroRNA microarray***

For analysis of TAZ-induced microRNAs, MG63 cells were transduced with empty vector or shTAZ. microRNAs were detected with High throughput sequencing technology. Differentially expressed microRNA transcripts with a p-value less than 0.01 and tweenty of the most differentially expressed miRNA were selected and the heatmap was drawn using Matlab.

**References**

1 Guo T, Lu Y, Li P *et al*. A novel partner of Scalloped regulates Hippo signaling via antagonizing Scalloped-Yorkie activity. *Cell research* 2013; 23:1201-1214.
2 Zhao B, Ye X, Yu J *et al*. TEAD mediates YAP-dependent gene induction and growth control. *Genes Dev* 2008; 22:1962-1971.

3 Ding S, Wu X, Li G, Han M, Zhuang Y, Xu T. Efficient transposition of the piggyback (PB) transposon in mammalian cells and mice. *Cell* 2005; 122:473-483.
4 Becam I, Rafel N, Hong X, Cohen SM, Milan M. Notch-mediated repression of bantam miRNA contributes to boundary formation in the *Drosophila* wing. *Development* 2011; 138:3781-3789.
5 Brennecke J, Hipfner DR, Stark A, Russell RB, Cohen SM. bantam encodes a developmentally regulated microRNA that controls cell proliferation and regulates the proapoptotic gene hid in *Drosophila*. *Cell* 2003; 113:25-36.
6 Zhang L, Ren F, Zhang Q, Chen Y, Wang B, Jiang J. The TEAD/TEF family of transcription factor Scalloped mediates Hippo signaling in organ size control. *Dev Cell* 2008; 14:377-387.

**Primers**

| Sr No | Name | Nucleotide sequence (5’-3’) |
| --- | --- | --- |
| 1 | pre-miR-224 | Ambion, PM12571 |
| 2 | Hsa-miR-224 Taqman Assay | ABI,4373187 |
| 3 | SMAD4 Forward Primer | aggatcagtaggtggaatag |
| 4 | SMAD4 Reverse Primer | tctaaaggttgtgggtctgc |
| 5 | β-actin Forward Primer | aggatcagtaggtggaatag |
| 6 | β-actin Reverse Primer | tctaaaggttgtgggtctgc |
| 7 | SMAD4-nhe-F | ctagctagcaccatggacaatatgtctattacg |
| 8 | SMAD4-not-R | atagtttagcggccgctcagtctaaaggtt |
| 9 | SMAD4-3’UTR-mut1-F | gtttggatatttttgtacttgatttgatgtactcttttttggtataatgtttaaatcatgtatg |
| 10 | SMAD4-3’UTR-mut1-R | catacatgatttaaacattataccaaaaaagagtacatcaaatcaagtacaaaaatatccaaac |
| 11 | SMAD4-3’UTR-mut2-F | ctttgccatcaatgatcatatcaattggcagtactcttgtatagagaatttaagtagaaaagttgcag |
| 12 | SMAD4-3’UTR-mut2-R | ctgcaacttttctacttaaattctctatacaagagtactgccaattgatatgatcattgatggcaaag |
| 13 | TAZ siRNA #1 | ctgcaacttttctacttaaattctctatacaagagtactgccaattgatatgatcattgatggcaaag |
| 14 | TAZ siRNA #2 | ctgcaacttttctacttaaattctctatacaagagtactgccaattgatatgatcattgatggcaaag |
| 15 | shTAZ | ggauacaggagaaaacgca |
| 16 | Lats1siRNA#1 | gaaccaaactctcaaacaa |
| 17 | Lats1 siRNA#2 | gcaagtcactctgctaatt |
| 18 | Lats2 siRNA#1 | gttcggaccttatcagaaa |
| 19 | Lats2 siRNA#2 | gatcggtgcctttggagaa |
| 20 | YAP1 siRNA#1 | ccaccaagcuagauaaaga |
| 21 | YAP1 siRNA#2 | ggucagagauacuucuuaa |
| 22 | shSMAD4 | gcatagtttgatgtgccatag |
