# Supplementary figures and images for: The TAZ–miR-224–SMAD4 axis promotes tumorigenesis in osteosarcoma
Source: Cell Death Dis. 2017 Jan 5;8(1):e2539–. doi: 10.1038/cddis.2016.468 (PMC5386375; doi:10.1038/cddis.2016.468)

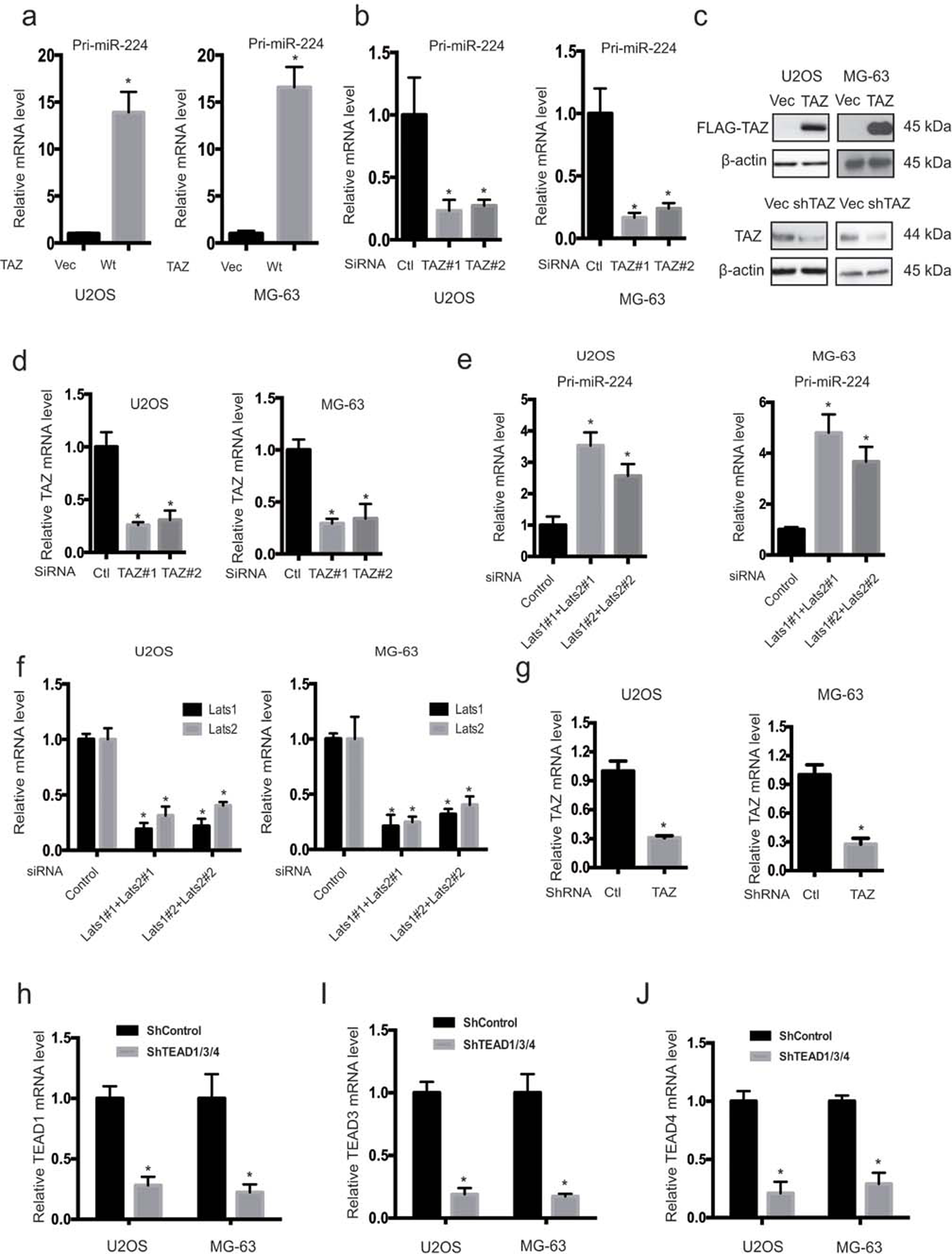

Supplement: Supplementary Figure 1 [file cddis2016468x3.tif]

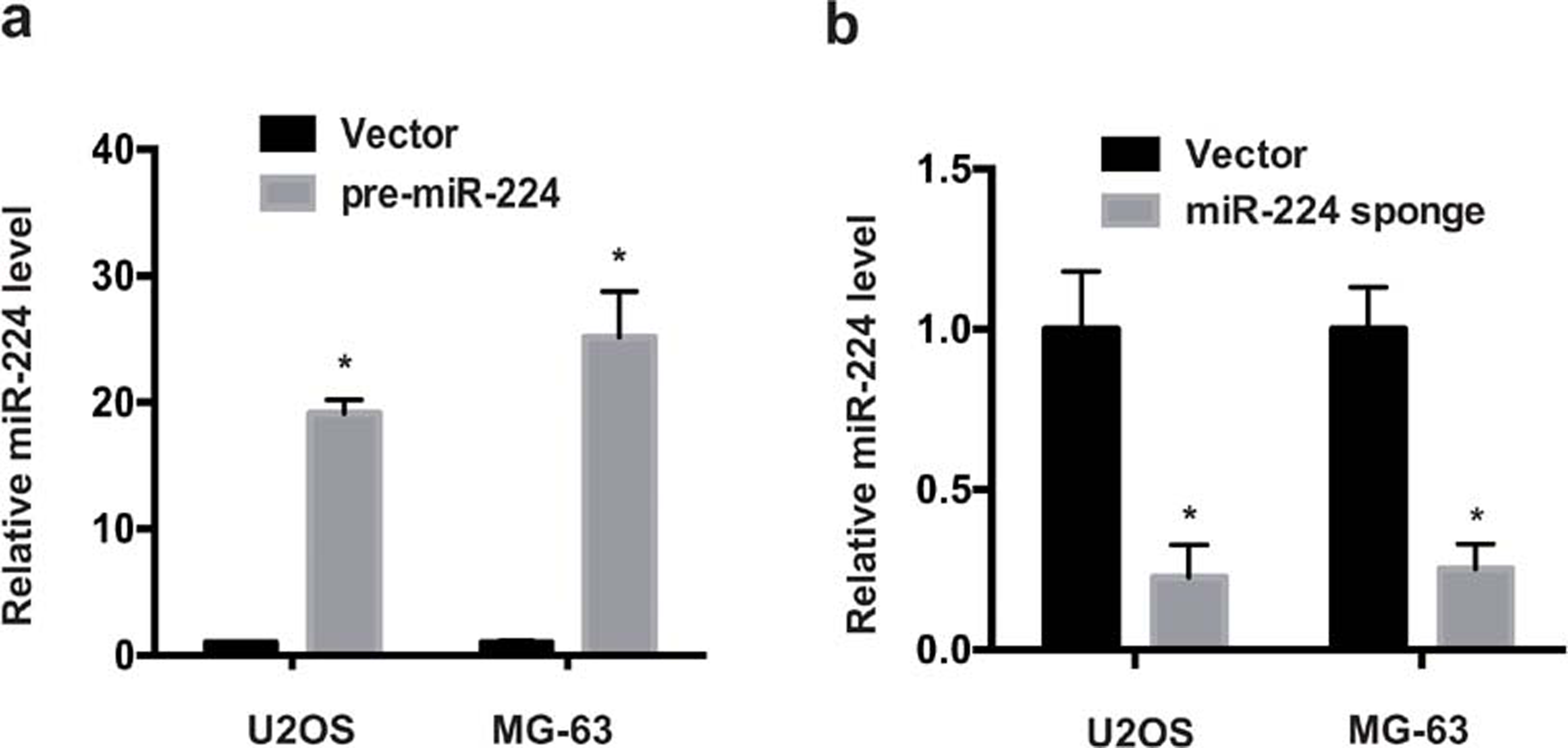

Supplement: Supplementary Figure 2 [file cddis2016468x4.tif]

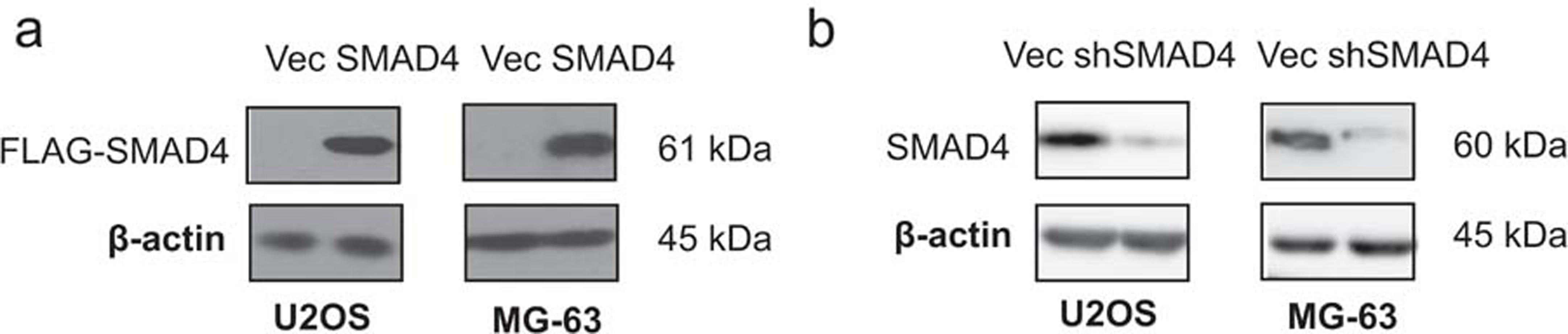

Supplement: Supplementary Figure 3 [file cddis2016468x5.tif]
